# Highly efficient green InP-based quantum dot light-emitting diodes regulated by inner alloyed shell component

**DOI:** 10.1038/s41377-022-00855-z

**Published:** 2022-05-30

**Authors:** Peng Yu, Sheng Cao, Yuliang Shan, Yuhe Bi, Yaqi Hu, Ruosheng Zeng, Bingsuo Zou, Yunjun Wang, Jialong Zhao

**Affiliations:** 1 School of Physical Science and Technology, MOE Key Laboratory of New Processing Technology for Non-ferrous Metals and Materials, Guangxi Key Laboratory of Processing for Non-Ferrous Metals and Featured Materials, Guangxi University, Nanning, 530004 China; 2 Suzhou Xingshuo Nanotech Co., Ltd. (Mesolight), Suzhou, 215123 China

**Keywords:** Inorganic LEDs, Photonic devices

## Abstract

InP-based quantum dot light-emitting diodes (QLEDs), as less toxic than Cd-free and Pb-free optoelectronic devices, have become the most promising benign alternatives for the next generation lighting and display. However, the development of green-emitting InP-based QLEDs still remains a great challenge to the environmental preparation of InP quantum dots (QDs) and superior device performance. Herein, we reported the highly efficient green-emitting InP-based QLEDs regulated by the inner alloyed shell components. Based on the environmental phosphorus tris(dimethylamino)phosphine ((DMA)_3_P), we obtained highly efficient InP-based QDs with the narrowest full width at half maximum (~35 nm) and highest quantum yield (~97%) by inserting the gradient inner shell layer ZnSe_x_S_1−x_ without further post-treatment. More importantly, we concretely discussed the effect and physical mechanism of ZnSe_x_S_1–*x*_ layer on the performance of QDs and QLEDs through the characterization of structure, luminescence, femtosecond transient absorption, and ultraviolet photoelectron spectroscopy. We demonstrated that the insert inner alloyed shell ZnSe_x_S_1−*x*_ provided bifunctionality, which diminished the interface defects upon balancing the lattice mismatch and tailored the energy levels of InP-based QDs which could promote the balanced carrier injection. The resulting QLEDs applying the InP/ZnSe_0.7_S_0.3_/ZnS QDs as an emitter layer exhibited a maximum external quantum efficiency of 15.2% with the electroluminescence peak of 532 nm, which was almost the highest record of InP-based pure green-emitting QLEDs. These results demonstrated the applicability and processability of inner shell component engineering in the preparation of high-quality InP-based QLEDs.

## Abstract (web summary)

In this work, the pure green-emitting InP/ZnSe_x_S_1−x_/ZnS quantum dots and their light-emitting diodes with high efficiency were successfully obtained by regulating the components of inner alloyed shell ZnSe_x_S_1−x_ layer.

## Graphical abstract

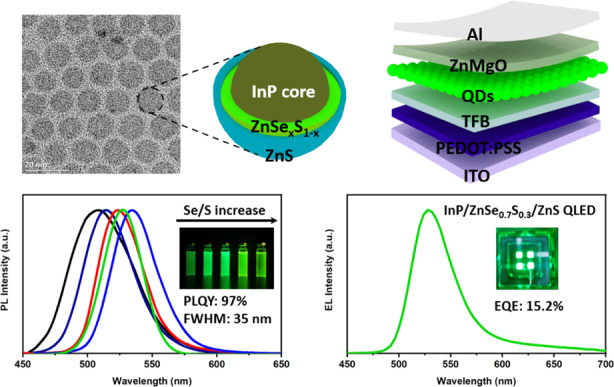

## Introduction

Quantum dot light-emitting diodes (QLEDs) have received considerable attention during these years due to their extraordinary color purity, reliability, cost-effectiveness, and efficiency^1–6^. Considering that about 15% of the global electricity was used on lighting, these advantages made QLEDs become the most promising substitute of traditional light-emitting diodes and organic light-emitting diodes for efficient lighting and display^7–12^. Nowadays, the cadmium chalcogenide-based quantum dots (QDs) and QLEDs have made great achievement which has already devoted in application;^13^ however, the intrinsic toxicity of Cd might inhibit their further development according to the restriction of hazardous substances directive^14–16^. In light of this, the indium phosphide (InP) QDs emerged as the most promising benign alternatives to heavy metal-free emitters^17,18^.

With continuous efforts and attempts, the electroluminescence (EL) spectra of InP-based QLEDs can be covered from 469 nm to 630 nm which almost covered the whole visible region^1,19–21^. Especially, the luminance of red-emitting InP-based QLEDs had reached 100,000 cd m^−2^ which was enough for outdoor displays and lighting. However, although the external quantum efficiency (EQE) of red-emitting InP-based QLEDs had reached 21.4% with considerable luminance which almost kept pace with Cd-based QLEDs, the green-emitting InP-based QLEDs still lagged behind while the EQE of Cd-based green-emitting QLEDs had already reached 23.9% in 2019^1,22^. As the most sensitive color of human eyes, it is of particular importance to improve the performance of green-emitting InP-based QLEDs for better lighting and display^23^. Therefore, the development of high-efficiency green InP-based QLEDs has become one of the foci of industry and academia.

It had been recognized that the broad full width at half maximum (FWHM) and low EQE of InP-based QLEDs was generally owing to the trapping of photoexcited charge on the QD interface defects^4,24,25^. The core-shell structure had been documented to be an effective strategy to suppress the interface defects. At present, the type I core-shell structure such as InP/ZnS has been widely adopted^26^. However, the large lattice mismatch between InP core and ZnS shell generated interface defects on the surface of QDs, thus making the QDs discourse low photoluminescence quantum yield (PLQY) and broad FWHM^24^. As a consequence, the design for the suitable gradient multiple core–shell structure aimed to decrease the interface stress and defects between the core and outer shell became an efficient method to enhance the performance of QDs and QLEDs^4,19,25–28^. For example, Shen’s group designed GaP inner shell aimed to reduce the lattice mismatch between InP core and ZnS outer shell to minimize the interfacial defects^24^. As a result, the obtained green-emitting InP/GaP/ZnS/ZnS QDs with about 70% PLQY and the EQE of their QLEDs reached 6.3%, 1.8 folds higher compared to the record at that time. However, the FWHM of 53 nm which was responsible for the low color purity still had a great space for further improvement. Although the GaP inner shell layer mitigated the lattice mismatch to a degree, there still existed a large mismatch between InP core and GaP layer. What’s more, the interface of InP core and inner shell layer influenced the PL properties of QDs very much which probably led to such a broad FWHM. Up to now, despite a lot of works on multi shell design in InP-based QDs, the optical performance of QDs was still not ideal, especially their FWHM was usually more than 35 nm^4^. An in-depth understanding of the relationship between the inner shell structure of QDs and luminescence performance was expected to be used to develop high-performance green-emitting InP-based QDs and QLED devices.

The poor performance of green-emitting InP-based QLEDs is also related to Auger recombination as a consequence of unbalanced injection between electrons and holes^29–31^. In order to balance the carrier injection in QLED, modifying the energy level position of the charge transport layer or QDs themselves was usually an effective way^30^. For example, Chae’s group tailored the highest occupied molecular orbital and lowest unoccupied molecular orbital level of electron transport layer with magnesium to reduce the electron mobility and thus balanced the injection of holes and electrons. As a result, the green-emitting InP-based QLED they made exhibited an EQE of 13.6% which was the first time that the EQE exceeded 10%^29^. Similarly, to reduce the mismatched mobility between electrons and holes, Chou et al. adopted ligand exchange strategy and modified the QDs surface with alkyl diamines which lifted the valence band (VB) and conduction band (CB) of InP QDs due to interface dipole interaction^32^. As a consequence, the holes’ mobility was facilitated whereas the electrons’ mobility was inhibited. The EL peak of QLED they fabricated was situated at 545 nm with an EQE of 16.3%. In order to give better scope to the superiority of QLEDs in display, the QLEDs with EL wavelength peak around 530 nm and high performance were required. As reported in Cd-based QLEDs^22^, adjusting the energy level position of QDs by tailoring inner alloyed shell components was also an effective method to promote the balanced charge injection in devices whereas it was still less reported in the InP-based QLEDs.

It should also be noted that most of the InP-based QDs nowadays were synthesized with expensive and hazardous phosphorus, tris(trimethylsilyl)phosphine ((TMS)_3_P)^1,14^. The highly active phosphorus indeed increased the potential dangers during the transport or operation process. Sun’s group synthesized QDs with considerable PLQY of 95% and FWHM of 45 nm based on tris(dimethylamino)phosphine ((DMA)_3_P) which was more economical and steadier compared to ((TMS)_3_P)^4,33–35^. Considering the needs of future industrial applications, using this environmentally friendly raw material of (DMA)_3_P to prepare InP-based QDs and systematically studying the relationship between multiple shell components and the performance of QLEDs was of great worthiness for further research. Herein, we reported the synthesis of highly efficient green-emitting InP/ZnSe_x_S_1−x_/ZnS QDs with an environmental phosphorus of (DMA)_3_P. We revealed the concrete physical mechanism about how the ZnSe_x_S_1−x_ inner shell layer influenced the performance of QDs and QLEDs through detailed characterizations such as steady-state and time-resolved PL spectroscopy, femtosecond transient absorption (TA), and ultraviolet photoelectron spectroscopy (UPS). These characterizations gave a deep insight of the effects on inserted inner alloyed shell ZnSe_x_S_1−x_ layer which provided bifunctionality to diminish the interface defects upon balancing the lattice mismatch and both lift the CB and VB of InP-based QDs to promote the balanced injection of the carriers. Through regulating the ratio between selenium and sulfur of the ZnSe_x_S_1−x_ inner shell, the as-synthesized QDs exhibited the narrowest FWHM of 35 nm and highest PLQY of 97%. Furthermore, the EQE of QLEDs we fabricated had reached 15.2%, which was close to the record of green-emitting InP-based QLEDs and was 2.15-folds higher than the record based on the same phosphorus. These results demonstrated that the luminescence performance of QDs and QLEDs can be significantly improved by the facile strategy, the inner shell component regulation. More importantly, our work gave reliable guidance for the designing of highly efficient green-emitting InP-based QDs and QLEDs which could advance the development of other QDs and QLEDs.

## Results

**Fig. 1:**
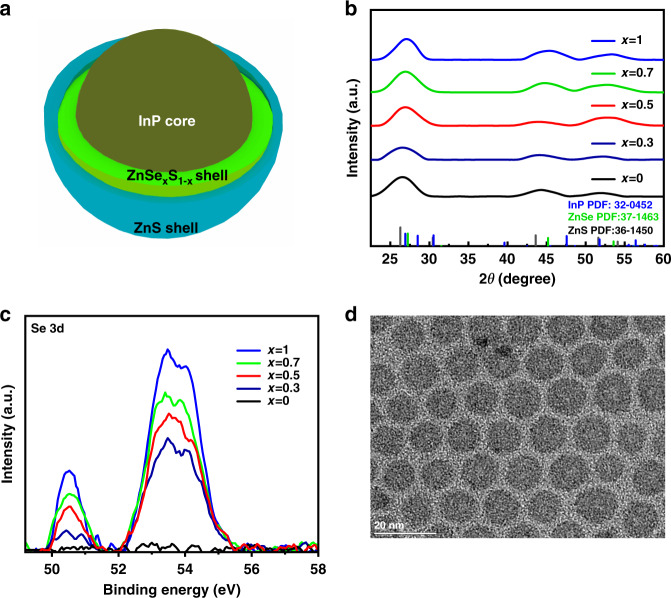
Structural and morphological properties of InP-based QDs. **a** Schematic diagram of InP/ZnSe_x_S_1−x_/ZnS structure. **b** XRD patterns for InP/ZnSe_x_S_1−x_ QDs. **c** XPS spectra of Se 3d regions of InP/ZnSe_x_S_1−x_ QDs. **d** TEM images of InP/ZnSe_0.7_S_0.3_/ZnS QDs

The environmental phosphorus of (DMA)_3_P was employed in the synthesis of InP-based QDs in consideration of the safety and the cost. Meanwhile, we noted that the large lattice mismatch between InP core and ZnS outer shell in InP/ZnS QDs not only led to large stress at the interface between them but also produced additional defects, and thus abated the optical properties of QDs^3^. To acquire high-quality InP-based QDs, we designed a gradient alloyed inner shell ZnSe_x_S_1−x_ layer between InP cores and outer ZnS shell as shown in Fig. 1a. The proportion of Se/S precursor used for inner shell growth was adjusted for the growth of ZnSe_x_S_1−x_ inner shell (see details in Supplementary Information). The InP/ZnSe_x_S_1−x_ QDs with uncoated outermost ZnS shells were systematically characterized to verify the effect of inner shell components on the composition and crystal structure of QDs. It was found that no impurity phase had been observed while changing the composition of the ZnSe_x_S_1−x_ inner shell, and the X-ray diffraction (XRD) patterns of InP/ZnSe_x_S_1−x_ fitted well with the standard InP, ZnSe, ZnS PDF cards (Fig. 1b). The lattice constants of InP and ZnSe_x_S_1−x_ obtained from Fig. 1b was shown in Table S1 (Supplementary Information). And the lattice spacing of ZnSe_0.7_S_0.3_ situated at the middle of InP core and ZnS outer shell indicates the balance of lattice mismatch which could facilitate the growth of ZnS outer shell. The X-ray photoelectron spectroscopy (XPS) of InP/ZnSe_x_S_1−x_ QDs showed that the In 3d, P 2p, and Zn 2p peaks were similar to the other works recorded before and barely affected by the feed ratio between Se and S precursors (Fig. S1 in Supplementary Information)^36,37^. The intensities of XPS peaks of Se 3d increased with the increasing feed ratio of Se/S as shown in Fig. 1c. The chemical component of ZnSe_x_S_1−x_ QDs was detected by energy dispersive spectrometer (Table S2 in Supplementary Information). The number of Se and S elements were not similar to the feed ratio yet remained coordinated tendency with it. It appeared that adjusting the proportion between Se and S precursors was a facile and efficient way to manipulate the inner shell structure without the phase transition of InP core.

The InP/ZnSe_x_S_1−x_/ZnS QDs were obtained with the further epitaxial growth of ZnS outer shell to improve their performance. It was found that the main peak of InP/ZnSe_x_S_1−x_/ZnS XRD patterns became narrower which proved the successful growth of ZnS outer shell (Fig. S2 in Supplementary Information). The morphology of InP/ZnSe_x_S_1−x_/ZnS QDs was also characterized by transmission electron microscopy (TEM). The TEM images illustrated that the single shell of ZnSe or ZnS was not sufficient to cover the InP cores which induced inhomogeneous shape of QDs (Fig. S3 in Supplementary Information). However, as we regulated the single shell to the gradient alloyed shell ZnSe_x_S_1−x_, the shape of QDs gradually became uniform and featured a spherical shape especially when *x* = 0.7 as shown in Fig. 1d. The average diameter of these QDs also gradually increased from 4.75 nm to 11.5 nm (Fig. S4 in Supplementary Information). These results indicated that the component-regulated inner ZnSe_x_S_1−x_ shell could facilitate the coating of the shell and promote the isotropic growth of InP-based QDs.

**Fig. 2:**
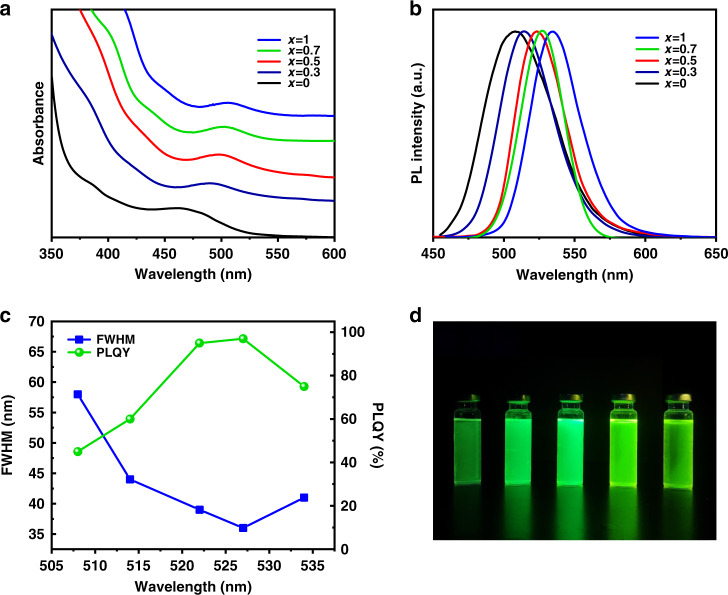
Optical properties of InP/ZnSe_x_S_1−x_/ZnS QDs (*x* = 0, 0.3, 0.5, 0.7, 1). **a**, **b** Absorption and PL spectra of various QDs. **c** FWHM-PL peak-PLQY of various QDs. **d** Digital photographs of QDs-solution with the same optical density (0.05) under 365 nm ultraviolet lamp (*x* = 0, 0.3, 0.5, 0.7, 1 from left to right)

Simultaneously, we revealed the effect of gradient alloyed inner shell ZnSe_x_S_1−x_ on the optical properties of InP/ZnSe_x_S_1−x_/ZnS QDs. As shown in Fig. 2a, the first exciton absorption peak shifted from 475 nm to 505 nm with varying different ratios between Se and S of the InP/ZnSe_x_S_1−x_/ZnS QDs. In addition, the PL peak also shifted from 508 nm to 535 nm as shown in Fig. 2b. It had been documented that the delocalization of electron wave function in InP core was sensitive to the ZnSe_x_S_1−x_ inner shell while the hole wave function was different^17^. The conduction band offset at the InP and ZnSe_x_S_1−x_ interface became smaller with an increasing ratio of Se/S which narrowed the bandgap of QDs and thus the first exciton and PL peak exhibited a red shift^17^. What’s more, the FWHM also gradually decreased to 35 nm when x = 0.7 and simultaneously the PLQY increased to 97% as shown in Fig. 2b and c. A great improvement, while the FWHM was 45 nm of green-emitting InP QDs based on the (DMA)3P reported by Sun’s group before and the FWHM (35 nm) of our QDs, was close to the state-of-the-art of green-emitting InP QDs (Table S3 in Supplementary Information)^25^. However, the FWHM increased again while the feed ratio of Se elements was further increased and the PLQY also decreased a little. This phenomenon was probably caused by Ostwald ripening (Fig. S3e in Supplementary Information) which the large size particles further increased, and the small size particles gradually shrank. The PLQY of as-synthesized InP/ZnSe_x_S_1−x_/ZnS QDs had good reproducibility (Fig. S5 in Supplementary Information). In addition, the digital photos of these QDs were shown in Fig. 2d. With the proper regulation of the inner ZnSe_x_S_1−x_ shell, the QDs apparently became brighter until *x* = 0.7. These results demonstrated that the PLQY and color purity of InP-based QDs could be effectively ameliorated by properly adjusting the composition of inner ZnSe_x_S_1−x_ shell.

To figure out how the inner shell components influenced FWHM and PLQY of InP/ZnSe_x_S_1−x_/ZnS QDs, the PL spectra were fitted with two Gaussian functions^38–40^. It was believed that the fitting curve in the high energy region with sharp FWHM originated from bandgap emission and the other curve in the lower energy region with broadened FWHM originated from trap emission generated from the interface defects between the core and inner shell layer^38^. And this kind of trap emission had been proved to be Auger recombination which was well documented before^39–42^. The trap emission ratio significantly decreased with the increasing ratio of Se/S of the ZnSe_x_S_1−x_ inner shell (*x* = 0–0.7) (Fig. S6 in Supplementary Information). When the amount of Se element was further increased, the trap emission ratio increased again (*x* = 1.0). As the consequence, the FWHM decreased first and then rose, and PLQY reversely.

**Fig. 3:**
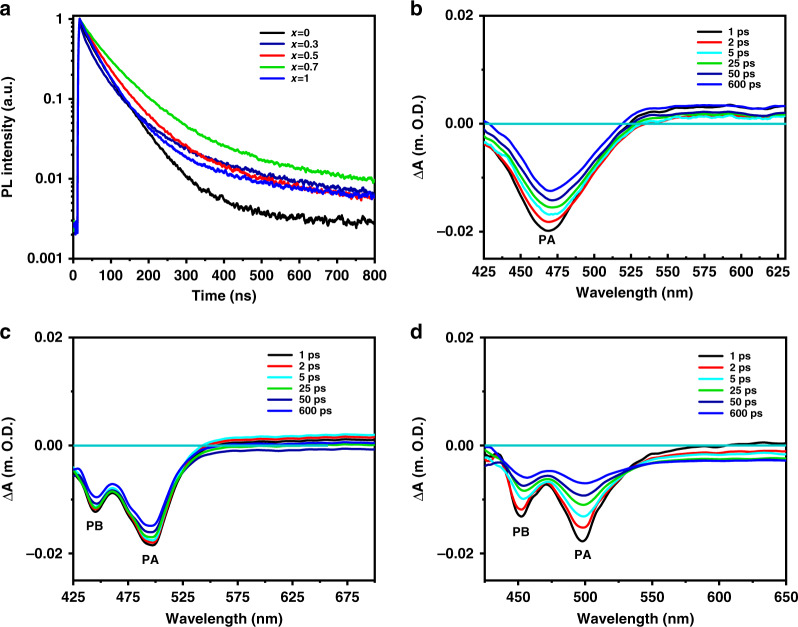
Time-resolved PL spectra and transient absorption spectra. **a** PL decay curves of various InP/ZnSe_x_S_1−x_/ZnS QDs. **b**–**d** TA spectra of the InP/ZnSe_x_S_1−x_/ZnS QDs (*x* = 0 (**b**), 0.7 (**c**), 1 (**d**))

Accordingly, the time-resolved PL spectroscopy was used to characterize the change in PL dynamics and consolidate our theory about how the inner shell components influenced FWHM and PLQY of QDs. As shown in Fig. 3a, the curves were fitted well with a double exponential function. In the light of Fermi golden rule, the PL lifetime was in reverse proportion with the overlap of electron and hole functions^43^. As a result, the fast decay process was considered as band-edge emission because of the large overlap of electrons and holes whereas the slow decay process was considered as trap emission^41,44^. With the increasing ratio of Se/S element of ZnSe_x_S_1−x_ inner shell, the ratio of trap/band-edge emission gradually decreased from 40% to 14% until *x* = 0.7, then the ratio of trap/band-edge emission rose again (Table S4 in Supplementary Information). Meanwhile, the average PL lifetime apparently increased from 44.20 ns to 76.80 ns and then decreased to 65.98 ns. These results coincided with the fitting curves as shown in Fig. S6 (Supplementary Information) and demonstrated that the appropriate ratio of Se/S element of ZnSe_x_S_1−x_ inner shell could reduce the trap emission and thus sharpen the FWHM and increase the PLQY.

To further understand the effect of inner shell components on the luminescence dynamics of our QDs, the TA spectra were employed, in which the delay time was prolonged from 1 ps to 600 ps (Figs. 3b–d, S7 in Supplementary Information). The obvious signal peak A (PA) was related to the 1S transition (1S_e_−1S_3/2_)^39^. The TA spectra showed similar feature of InP/ZnSe_x_S_1−x_/ZnS QDs as the bleach peaks (PA) shifted from 473 nm to 503 nm with the manipulated feed ratio of Se and S, which were almost consistent with the steady-state absorption spectrum. We attributed the other apparent peak B (PB) to the interface between InP core and ZnSe_x_S_1−x_ inner shell while the pure InP/ZnS/ZnS QDs showed noncorresponding signal^40^. As shown in Fig. 3c, the TA spectra of InP/ZnSe_0.7_S_0.3_/ZnS exhibited a slight shift of peaks for both PA and PB, which confirmed the restrain of the interface defects and featured layered quantum-well structure^41,45–47^. While the inner shell was tuned to InP/ZnSe/ZnS QDs as shown in Fig. 3d, the peaks showed apparently bleach at PB, indicating the weakened passivation ability of the traps at InP core and ZnSe interface^45–47^. What’s more, the dynamic spectra of these samples were characterized as shown in Fig. S8 (Supplementary Information). The fast-trapping process (~10 ps) increased with the increasing amount of Se elements in the inner shell layer and reduced the trap states until *x* = 0.7. And the trapping time became faster of pure ZnSe inner shell layer implying the increasing trapping state. These phenomena thus further indicated that the appropriate inner shell components could reduce the interface defects between the InP core and ZnSe_x_S_1−x_ inner shell.

**Fig. 4:**
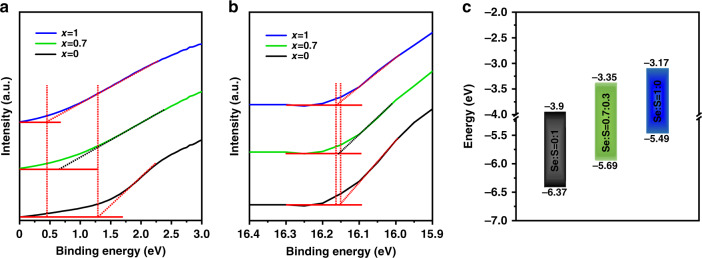
The energy level of InP/ZnSe_x_S_1−x_/ZnS QDs. UPS spectra of **a** the valence-band edge regions and **b** the secondary cutoff regions of InP/ZnSe_x_S_1−x_/ZnS QDs (*x* = 0, 0.7, 1). **c** Energy level diagram of corresponding QDs

To ulteriorly reveal the influence of inner shell components on the energy level structure of InP/ZnSe_x_S_1−x_/ZnS QDs, the UPS spectra of these QDs were characterized. We picked three typical samples with different inner shell components, InP/ZnS/ZnS, InP/ZnSe/ZnS along with the InP/ZnSe_0.7_S_0.3_/ZnS sample which had the best optical performance among the samples for comparison. Figure 4a and b shows the UPS spectra of the valence-band edges and secondary cutoff regions of InP/ZnSe_x_S_1−x_/ZnS QD film. The valence-band maximum (VBM) of these samples was calculated with the following equation: VBM = *hν* − (*E*_cutoff_ − *E*_cutonset_), where the *E*_onset_ was the onset energy in the valence band region and the *E*_cutoff_ represented the high binding energy cutoff^48^. The calculated VBM positions of InP/ZnS/ZnS, InP/ZnSe_0.7_S_0.3_/ZnS, InP/ZnSe/ZnS QDs were −6.95, −5.70, and −5.49 eV, respectively. Furthermore, the conduction-band minimum (CBM) was calculated with the value of VBM, and the bandgap which was obtained from the absorption spectra, respectively (Fig. S9 in Supplementary Information). The as-calculated CBM positions of InP/ZnS/ZnS, InP/ZnSe_0.7_S_0.3_, and InP/ZnSe/ZnS were −3.9, −3.35, and −3.17 eV, respectively. As shown in Fig. 4c, the band alignments of QDs rose along with the increasing amount of Se elements. Considering that the poor performance of InP-based QLEDs was usually caused by the over injection of electrons, the increasing position of VBM and CBM was expected to facilitate the hole injection, promote the balance of carrier injection and then improve the performance of QLEDs^26,29^.

**Fig. 5:**
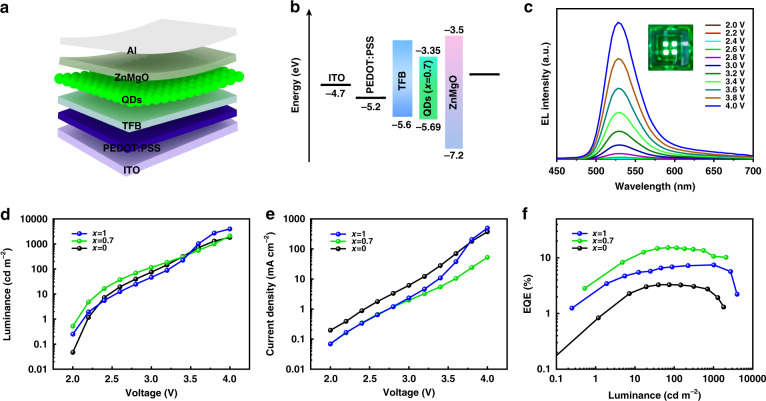
Schematic of the device structure and EL performance of the InP/ZnSe_x_S_1−x_/ZnS (*x* = 0, 0.7, 1) QLEDs. **a** Schematic of various layers in the device structure. **b** Energy band diagram of green-emitting InP/ZnSe_0.7_S_0.3_/ZnS QLEDs. **c** EL spectra. **d** Current density−voltage (*J*−*V*) characteristics. **e** Luminance−voltage (*L*−*V*) characteristics. **f** EQE as a function of luminance

As proof of concept, these InP-based QDs with different inner ZnSe_x_S_1−x_ shell components were used to construct QLED devices to better judge the potential applications. The QLED with multiple layers were disposed in the following order: a patterned indium tin oxide glass (ITO), poly(ethylene dioxythiophene):polystyrene sulfonate (PEDOT:PSS) (45 nm), poly(9,9- dioctylfluorene-co-N-(4-(3-methylpropyl))diphenylamine) (TFB) (40 nm), QDs (25 nm), ZnMgO (60 nm), and Al cathode (100 nm) as shown in Fig. 5a and the corresponding energy band diagram of InP/ZnSe_0.7_S_0.3_/ZnS QLEDs was illustrated in Fig. 5b. The EL spectra of QLEDs with the emissive layer of InP/ZnSe_0.7_S_0.3_/ZnS QDs demonstrated that the EL peaks are all situated at 532 nm with a FWHM of 45 nm under driven voltage between 2 and 4 V (Fig. 5c and Fig. S10 in Supplementary Information). Compared to the PL spectrum of QDs in solution, the shifted EL peak and broadened FWHM were probably due to the electric field-induced Stark effect and Forster resonance energy transfer^33,49^. At the same time, we selected the QLEDs with the emissive layer of InP/ZnS/ZnS, InP/ZnSe_0.7_S_0.3_/ZnS, and InP/ZnSe/ZnS QDs as a demonstration. In consideration of the high voltage might damage the structure of QLEDs, we selected the voltage region from 2 to 4 V, which was enough to characterize the performance of QLEDs as the other works had done before^50,51^. The luminance−voltage (*L–V*) and current density−voltage (*J–V*) curves were shown in Fig. 5d and e. The maximum luminance of these QLEDs (*x* = 0, 0.7, 1) at 4 V were 1900, 2300, and 3057 cd m^−2^, respectively, which almost transcended the record up to date. The QLEDs based on InP/ZnS/ZnS and InP/ZnSe/ZnS QDs acquired the highest EQE of 3.1% and 8.6% as shown in Fig. 5f, respectively. Remarkably, the QLEDs with the InP/ZnSe_0.7_S_0.3_/ZnS QDs emissive layer exhibited terrific EQE of 15.2% which was closed to the state-of-the-art of the green-emitting InP-based QLEDs as shown in Table S5 (Supplementary Information). The preparation of this high-performance QLED also had high reproducibility. (Fig. S11 in Supplementary Information). Such an improvement of the EQE among these QLEDs possibly results from the gradient inner shell structure decreased the interface defects and lifted the VB and CB energy level which promote the balanced injection of carriers. These results indicated that it was feasible to regulate the inner shell components and the gradient alloyed inner shell could significantly improve the performance of QLEDs.

## Discussion

In conclusion, we obtained the highly efficient green-emitting InP/ZnSe_x_S_1−x_/ZnS QDs based on an environmental phosphorus of (DMA)_3_P. Through regulation of the inner shell components, the as-obtained green-emitting InP-based QDs had a narrow FWHM of 35 nm and high PLQY of 97%. The X-ray diffraction, time-resolved PL, and femto-transient absorption spectroscopy showed that the gradient alloyed inner shell layer ZnSe_x_S_1−x_ reduced the lattice mismatch, diminished the interface defects and trap emission. Meanwhile, it was discovered that the component engineering of the inner shell could tailor the energy level position of QDs, which improve the balanced carrier injection in QLEDs. As proof of concept, these InP-based QDs with different inner ZnSe_x_S_1−x_ shell components were used to construct QLED devices and exhibited extraordinary performance with the highest EQE of 15.2%, which was close to the state-of-the-art and was about twofolds higher than the QLEDs based on the same phosphorus. It is believed that the present strategy can promote the further development and the application of green-emitting InP-based QDs and QLEDs and give a reliable guidance for the designing of the other highly efficient QDs and QLEDs.

## Materials and methods

### Chemicals

Indium chloride (InCl_3_), zinc chloride (ZnCl_2_), zinc iodide (ZnI_2_), zinc acetate (Zn(OAc)_2_), sulfur powder (S), selenium powder (Se), trioctylphosphine (TOP), oleylamine (OLA), zinc stearate (Zn(st)_2_), 1-octadecene (ODE), 1-dodecanethiol (DDT), tris[2-(diphenylphosphino)ethyl]phosphine were purchased from Aladdin. PEDOT:PSS, poly[(9,9-dioctylfluorenyl-2,7-diyl)-co-(4,4′-(N-(4-sec-butylphenyl)diphenylamine)) (TFB, molecular weight 300,000), and zinc oxide (ZnMgO) were purchased from Suzhou Xingshuo Nanotech Co., Ltd. All chemicals were used without any purification.

### Precursor preparation

The zinc precursor (0.5 M) was made by dissolving 250 mmol Zn(st)_2_ in 400 mL ODE at 120 ^o^C under vacuum. The other zinc precursor (0.5 M) was made by adding 125 mmol Zn(OAc)_2_ in 250 mL OLA and then heating the flask to 70 ^o^C for 1 h under vacuum for later use. The TOP-Se solution (2 M) was made by adding 500 mmol pure Se powder in 250 mL TOP. The TOP-S solution (2 M) was made by adding 500 mmol pure S powder in 250 mL TOP.

### Synthesis of InP/ZnSe_x_S_1−x_/ZnS QDs

In a typical synthesis scheme, 1 mmol InCl_3_, 0.1 mmol ZnCl_2_, 5 mmol ZnI_2_, and 10 mL OLA were added into a 250 mL three-neck flask bottle. The mixture was degassed at 120 ^o^C for 1 h. Then the temperature was increased to 180 ^o^C under Ar flow. Inject 0.9 mL DMA_3_P into the flask and keep for 30 min. Increased the temperature to 200 ^o^C, then 2 mL ZnCl_2_-OLA was added. Then 20 mL Zn(st)_2_ was added. 3.5 mL TOP-Se solution was injected at 3.5 mL h^−1^ and 1.5 mL TOP-S solution was injected at 1.5 mL h^−1^ at 240 ^o^C to grow ZnSe_0.7_S_0.3_ inner shell. For the different components of the ZnSe_x_S_1−x_ inner shell, the TOP-Se and TOP-S ratio was just changed at 240 ^o^C. The temperature was increased to 310 ^o^C and kept for another 30 min. Add 20 mL Zn(st)_2_ and inject 4 mL TOP-S at 4 mL h^−1^ when the temperature becomes steady at 310 ^o^C. Then the flask was cooled down to 240 ^o^C, then 20 mL Zn(OAc)_2_ at 10 mL h^−1^ and 7 mL TOP-S at 3.5 mL h^−1^ were injected. After the reaction, the flask was cooled down to room temperature and the samples were dispersed in hexane with the ratio 1:1 and were centrifuged at 10,310 rpm for 5 min. Keep the supernatant solution redispersed in ethanol with a ratio of 1:3. Then the solution was centrifuged at 10,310 rpm for 5 min. The supernatant solution was discarded. The precipitation was dispersed in 10 mL heptane for further characterization.

### Fabrication of InP-based QLEDs

The QLEDs were fabricated based on the structure of ITO/PEDOT:PSS/TFB/QDs/ZnO/Al (100 nm). All these layers were spin-coated onto ITO glasses except for Al cathode. As for Al cathode, we deposited it through thermal evaporation methods under vacuum. ITO glasses were cleaned with washing water, deionized water, acetone, and isopropanol under ultrasonication successively for 30 min. Then the ITO glasses were put into ultraviolet ozone cleaning machine for 10 min to finish the last cleaning process. PEDOT:PSS (4083) were spin-coated onto ITO glasses at 3000 rpm for 40 s and were heated at 130 °C for 20 min. Then the prepared TFB solution (8 mg mL^−1^ dissolved in chlorobenzene), QDs solution (20 mg mL^−1^ in octane) were spin-coated layer by layer in the glove box at 3000 rpm for 40 s and subsequently were heated at 130 ^o^C for 20 min and at 80 ^o^C for 5 min respectively. Then the ZnO solution (27 mg mL^−1^ in ethanol) was spin-coated at 3000 rpm for 30 s and was heated at 80 ^o^C for 10 min. Finally, the devices were transformed to vacuum coating machine under a vacuum level of 5 × 10^−4 ^Pa for the deposition of Al cathode with the speed of 5 nm s^−1^. The patterned area of the devices is 4 mm^2^. All devices were encapsulated in the glove box through commercially available ultraviolet-curable resin for further characterization.

### Characterization

The X-ray diffraction pattern was measured using a Cu K*α* (*λ* = 1.5405 Å) radiation rotating anode and an X-ray diffractometer (model: SMARTLAB 3 KW) manufactured by Rigaku Corporation in the 2*θ* range of 20–80^o^. TEM images were obtained using a JEOL JEM 2100PLUS microscope operating at 200 kV. The samples were prepared by dropping the diluted QDs onto carbon coated 200 mesh copper grids.

The absorption spectra and PL spectra of the samples dispersed in hexane were characterized with a UV–vis spectrophotometer (PerkinElmer Instruments, Lambda 750) and Horiba Fluorolog®-3 systems, using the Synapse PLUS CCD (Charge Coupled Device) detection system and Xenon lamp (450 W). The excitation wavelength of Xenon lamp was 450 nm for these samples with both slit widths of 1 nm. The Horiba Fluorolog^®^-3 systems were further used to calculate the quantum yield for samples in solution, using the F-3029 integrating sphere accessory, the Quanta-φ and Xenon lamp (450 W). The slit width was fixed at 3 nm and the data was accumulated 4 times. We prepared each QD in different batches according to the same recipe to confirm the reproducibility of the synthetic process. The TRPL spectra were also characterized based on Fluorolog^®^-3 and related accessories. The sample was excited by a pulsed laser (Horiba Delta Diode DD-405L). Acquisition of the emission signal is synchronized to the pulse, with specified delay and sampling times, to produce time-resolved spectral data which collected by delta diodes. The PL emission from the samples was collected by a pair of lenses into the concave holographic grating of 600 g mm^−1^ and detected by photomultiplier tube. The Femtosecond transient absorption spectra were characterized with a femtosecond laser system containing solid-state titanium gem, which is produced by American Coherence Company. The center wavelength of the output fundamental frequency light is 800 nm, the pulse width is 130 fs, the single pulse energy is 3.41 mJ, and the repetition rate is 1 kHz. During the test, the femtosecond laser with a repetition rate of 1 kHz and wavelength of 800 nm is divided into two beams (9:1). Among them, the larger beam multiplies the frequency of 800 nm laser to 400 nm through BBO frequency doubling crystal. The XPS and UPS spectra of InP/ZnSe_x_S_1−x_/ZnS QDs were investigated via X-ray photoelectron spectrometer (ESCALAB 250XI+). For the UPS and XPS measurements, the samples were prepared by spin casting the different InP/ZnSe_x_S_1−x_/ZnS QDs onto ITO glass substrates. All QLEDs were characterized under ambient conditions. The current density-voltage (*J–V*) characteristics for the QLED devices were measured using a Keithley 2400 source meter. The luminance of the QLED devices was measured with a well-calibrated spectral scanning colorimeter (Photo Research 655). The electroluminescence spectra were measured using a spectrometer (Ocean optics, QE65000) and a Keithley 2400 source meter. The EQE was calculated according to the formula *η* = $\frac{{{\uppi Le}}}{{{{{\mathrm{KmhcJ}}}}}}$*$\frac{{{\int} {{{{\mathrm{I}}}}\left( \lambda \right)\lambda {{{\mathrm{d}}}}\lambda } }}{{{\int} {{{{\mathrm{I}}}}\left( \lambda \right){{{\mathrm{V}}}}\left( \lambda \right){{{\mathrm{d}}}}\lambda } }}$, where *e* is the electron charge, *h* is the Planck constant, *c* is the velocity of light and Km = 683 lm W^−1^ is the maximum luminous efficacy. *J* is current density, *I(λ)* is the relative electroluminescence intensity at wavelength *λ*, *V*(*λ*) is the normalized photonic spectral response function, and *L* is the total luminance.

## Supplementary information

Supplementary information

Confidential Certificate

Figure 1-bitmap

Figure 2-bitmap

Figure 5-bitmap

## Data Availability

All data supporting the findings of this study are available within the paper and its Supplementary Information
