# Supplementary material for: Highly efficient green InP-based quantum dot light-emitting diodes regulated by inner alloyed shell component
Source: Light Sci Appl. 2022 May 30;11:162. doi: 10.1038/s41377-022-00855-z (PMC9151710; doi:10.1038/s41377-022-00855-z)
Supplement: Supplementary file 1 — Supplementary information [file 41377_2022_855_MOESM1_ESM.docx]

**Supplementary Information for**

**Highly efficient green InP-based quantum dot light-emitting diodes regulated by inner alloyed shell component**

Peng Yu, ^1^ Sheng Cao, ^*, 1^ Yuliang Shan ^2^, Yuhe Bi, ^1^ Yaqi Hu, ^1^ Ruosheng Zeng, ^1^ Bingsuo Zou, ^1^ Yunjun Wang, ^*, 2^ and Jialong Zhao^*, 1^

1 School of Physical Science and Technology, MOE Key Laboratory of New Processing Technology for Non-ferrous Metals and Materials, Guangxi Key Laboratory of Processing for Non-ferrous Metals and Featured Materials, Guangxi University, Nanning 530004, China.

2 Suzhou Xingshuo Nanotech Co., Ltd. (Mesolight), Suzhou 215123, China.

*Correspondence: [caosheng@gxu.edu.cn](mailto:caosheng@gxu.edu.cn); [yjwt@mesolight.cc](mailto:yjwt@mesolight.cc); zhaojl@gxu.edu.cn


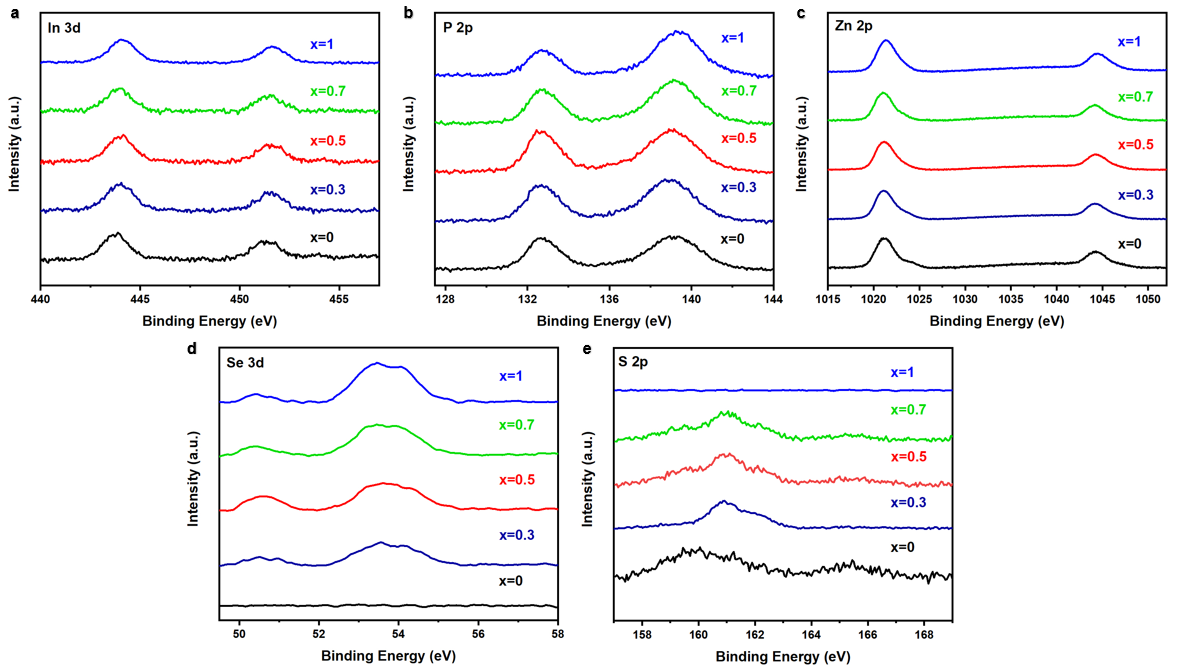


**Fig. S1.** Experimental XPS spectra of InP/ZnSe_x_S_1-x_ QDs (x=0, 0.3, 0.5, 0.7, 1). (a) In 3d. (b) P 2p. (c) Zn 2p. (d) Se 3d. (e) S 2p.

**Table S1.** Lattice constants of InP and InP/ZnSe_x_S_1-x_ QDs

| Sample | Lattice constant (Å) |
| --- | --- |
| InP | 5.72 |
| InP/ZnSe | 5.67 |
| InP/ZnSe_0.7_S_0.3_ | 5.55 |
| InP/ZnSe_0.5_S_0.5_ | 5.49 |
| InP/ZnSe_0.3_S_0.7_ | 5.45 |
| InP/ZnS | 5.40 |

**Table S2.** Chemical compositions of InP/ZnSe_x_S_1-x_ QDs synthesized with different proportion of Se/S precursor.

| Element  (atomic %) | In | P | Zn | Se | S |
| --- | --- | --- | --- | --- | --- |
| InP/ZnS | 16.38 | 33.20 | 35.76 | 0 | 14.66 |
| InP/ZnSe_0.3_S | 14.24 | 28.04 | 31.35 | 16.97 | 9.40 |
| InP/ZnSe_0.5_S_0.5_ | 12.76 | 30.10 | 29.91 | 19.31 | 7.92 |
| InP/ZnSe_0.7_S_0.3_ | 12.57 | 35.79 | 25.74 | 19.26 | 6.64 |
| InP/ZnSe | 10.96 | 23.47 | 33.98 | 31.59 | 0 |


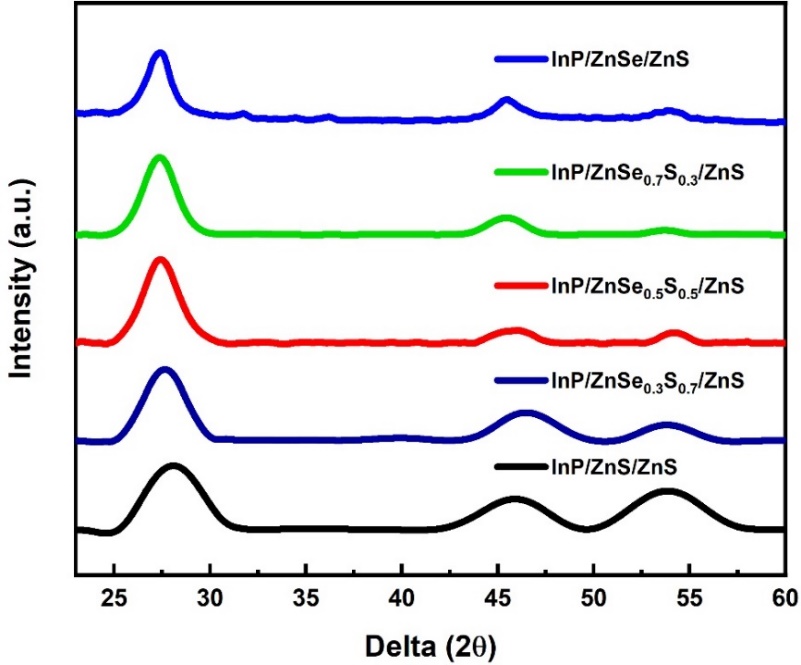


**Fig. S2.** XRD patterns of InP/ ZnSe_x_S_1-x_/ZnS QDs (x=0, 0.3, 0.5, 0.7, 1).


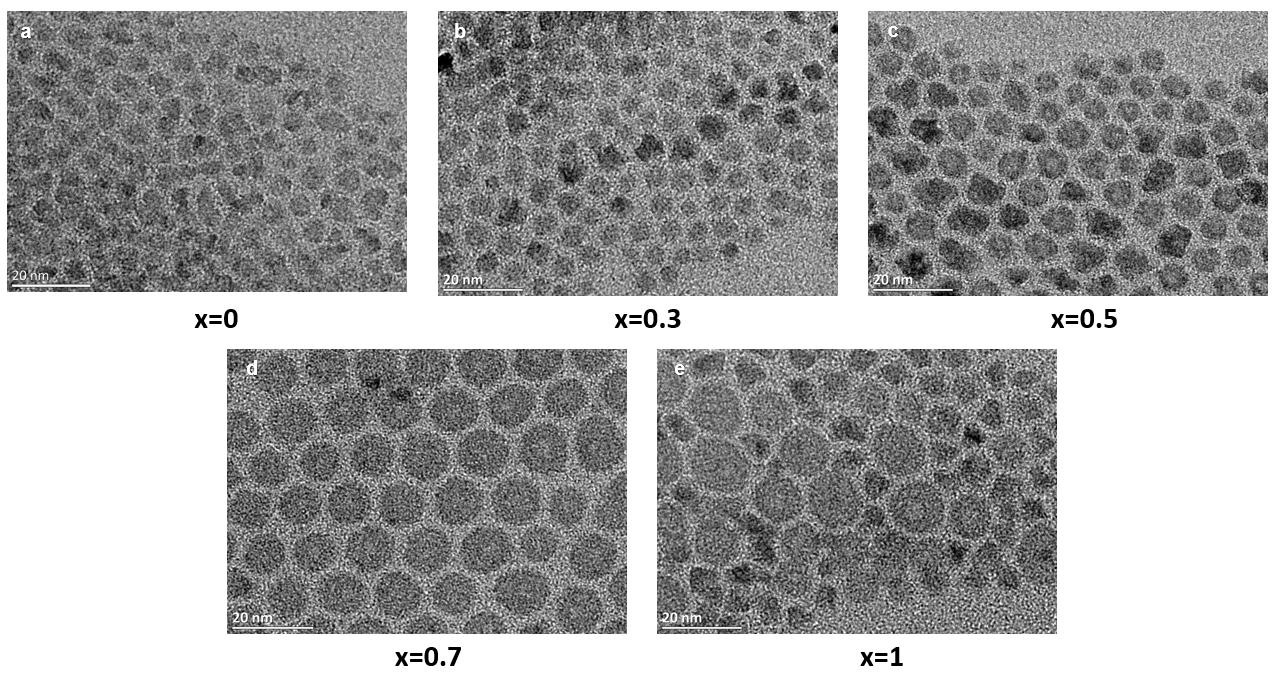


**Fig. S3.** TEM images of InP/ZnSe_x_S_1-x_/ZnS QDs (x=0, 0.3, 0.5, 0.7, 1).


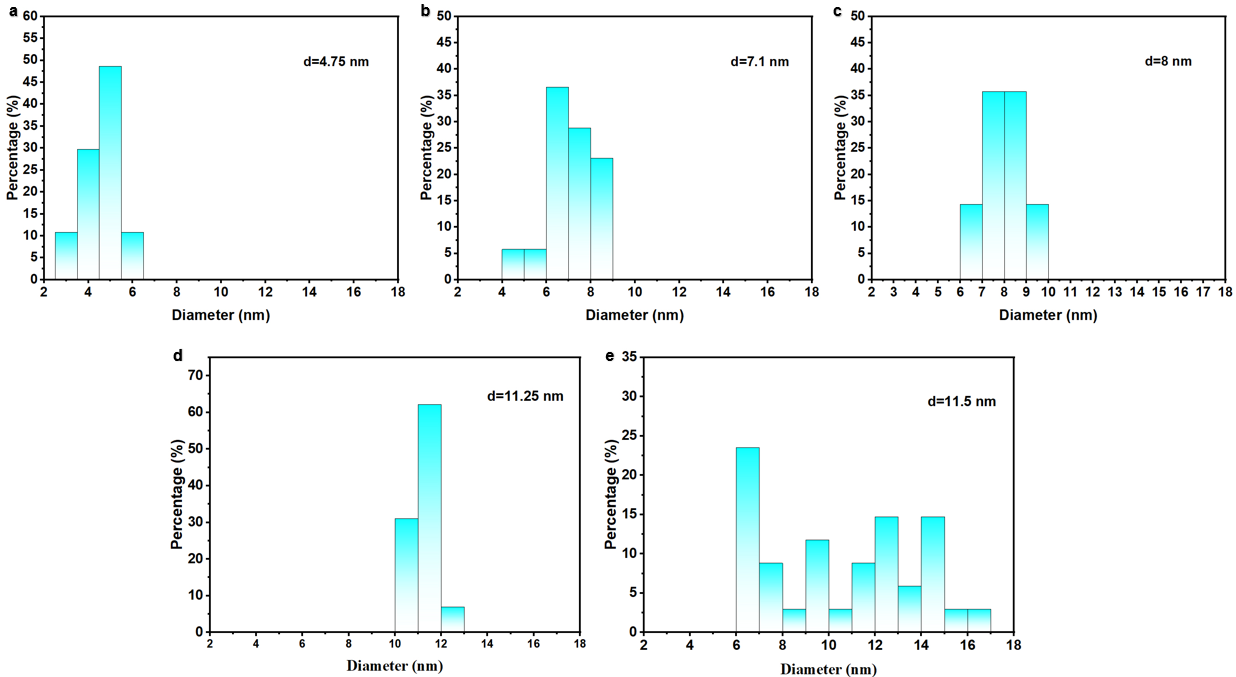


**Fig. S4.** The size histogram of InP/ZnSe_x_S_1-x_/ZnS QDs (a) x=0, (b) x=0.3, (c) x= 0.5, (d) x=0.7, (e) x=1.


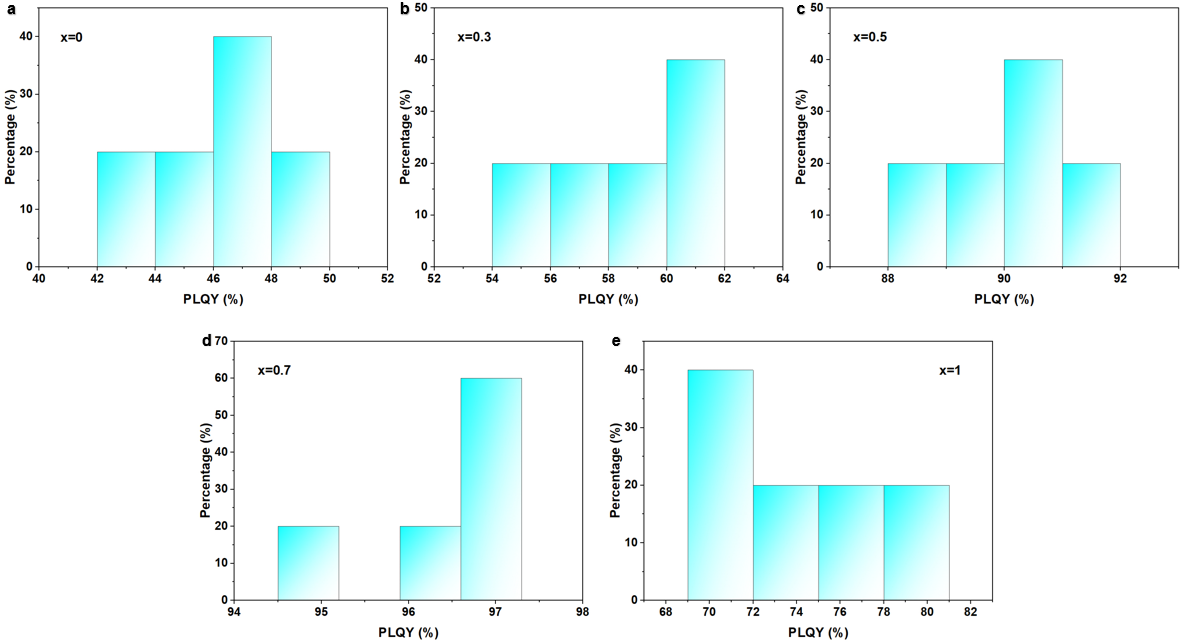


**Fig S5.** Reproducibility for the PLQY of the InP/ZnSe_x_S_1-x_/ZnS QDs (a) x=0, (b) x=0.3, (c) x=0.5, (d) x=0.7, (e) x=1.

**Table S3**. PL properties of state-of-art InP-based QDs.

|  | Phosphorus precursors | PL peak (nm) | FWHM (nm) | PLQY (%) | Ref. |
| --- | --- | --- | --- | --- | --- |
| 2018 | (TMS_3_)_3_P | 533 | 37 | 67 | 1 |
| 2019 | (TMS_3_)_3_P | 528 | 36 | 95 | 2 |
| 2019 | (TMS_3_)_3_P | 516 | 53 | 80 | 3 |
| 2019 | —— | 531 | 34 | 82 | 4 |
| 2020 | (TMS_3_)_3_P | 537 | 43 | 82 | 5 |
| 2020 | —— | 525 | 40 | 81 | 6 |
| 2020 | DMA_3_P | 527 | 37 | 87 | 7 |
| 2021 | DMA_3_P | 518 | 46 | 67.5 | 8 |
| 2021 | DMA_3_P | 510 | 45 | 95 | 9 |
| 2022 | DMA_3_P | 528 | 35 | 97 | This work |


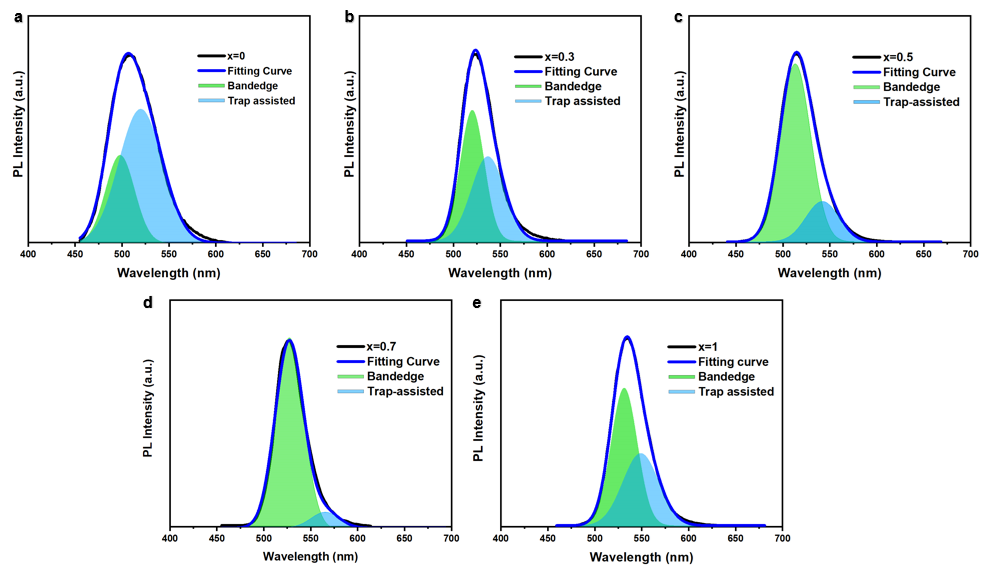


**Fig. S6.** Fitted PL spectra of InP/ZnSe_x_S_1-x_/ZnS QDs (x=0, 0.3, 0.5, 0.7, 1) with two Gaussian line shapes.

**Table S4.** The PL decay parameters of InP/ZnSe_x_S_1-x_/ZnS QDs (x=0, 0.3, 0.5, 0.7, 1).

|  | **x=0** | **x=0.3** | **x=0.5** | **x=0.7** | **x=1** |
| --- | --- | --- | --- | --- | --- |
| PLQY | 45% | 57% | 91% | 95% | 78% |
| A_1_,τ_1_ | 0.6, 23 ns | 0.65, 26 ns | 0.82, 48.8 ns | 0.86, 53.9 ns | 0.74, 56.67 ns |
| A_2_,τ_2_ | 0.4, 76 ns | 0.35, 93 ns | 0.18, 138.8 ns | 0.14, 138.9 ns | 0.26, 138.9 ns |
| τ_avg_ | 44.20 ns | 49.45 ns | 65 ns | 76.8 ns | 65.98 ns |

The PL decay curves of QDs were all fitted by a double-exponential function:

*y*=$A_{1}*\exp\left( -\frac{i}{\tau_{1}} \right)+A_{2}*exp(-\frac{i}{\tau_{2}})$ ……………(2)

where A_i_ and *τ_i_* are amplitudes and decay times of components (i=1, 2). The average lifetime (*τ_avg_*) was calculated by the following equation:

*τ_avg_*=$\frac{A_{1}*{\tau_{1}}^{2}+A_{2}*{\tau_{2}}^{2}}{A_{1}*\tau_{1}+A_{2}*\tau_{2}}$…………………………..………(3)


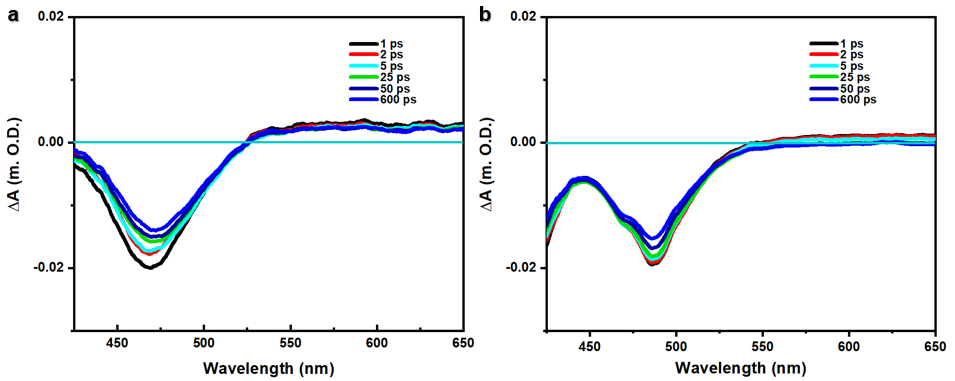


**Fig. S7.** TA spectra of (a) InP/ZnSe_0.3_S_0.7_/ZnS and (b) InP/ZnSe_0.5_S_0.5_/ZnS QDs.

**
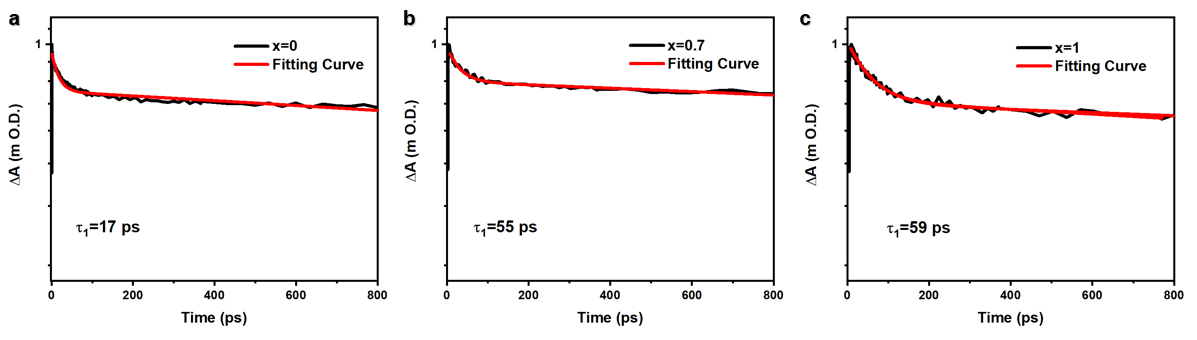
**

**Fig. S8.** Dynamic spectra of InP/ZnSe_x_S_1-x_/ZnS QD solutions (x=0, 0.7, 1).


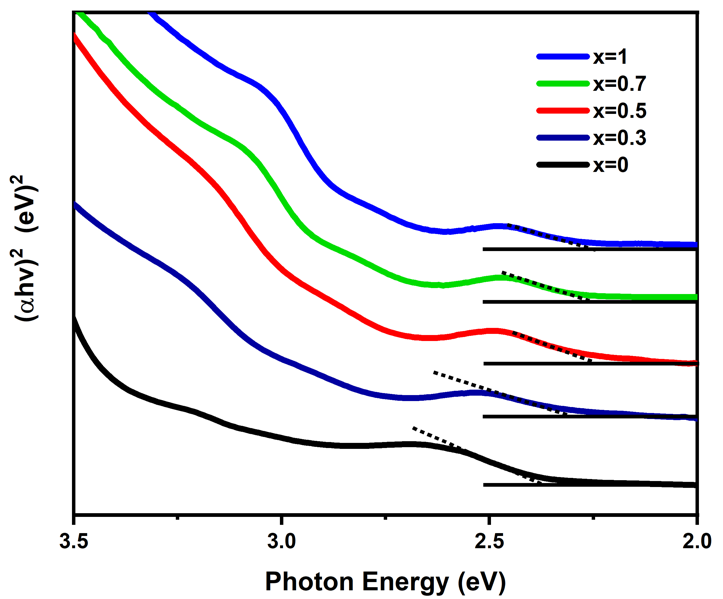


**Fig. S9.** Absorption spectra of InP/ZnSe_x_S_1-x_/ZnS QD films (x=0, 0.3, 0.5, 0.7, 1).


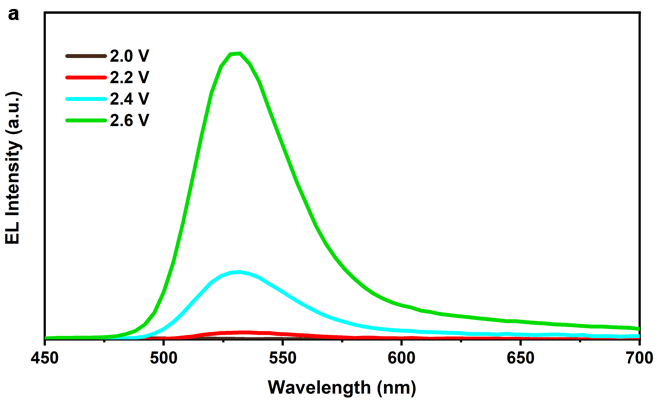


**Fig. S10.** Magnified EL spectra of InP/ZnSe_0.7_S_0.3_/ZnS QLEDs at 2.0-2.6 V.


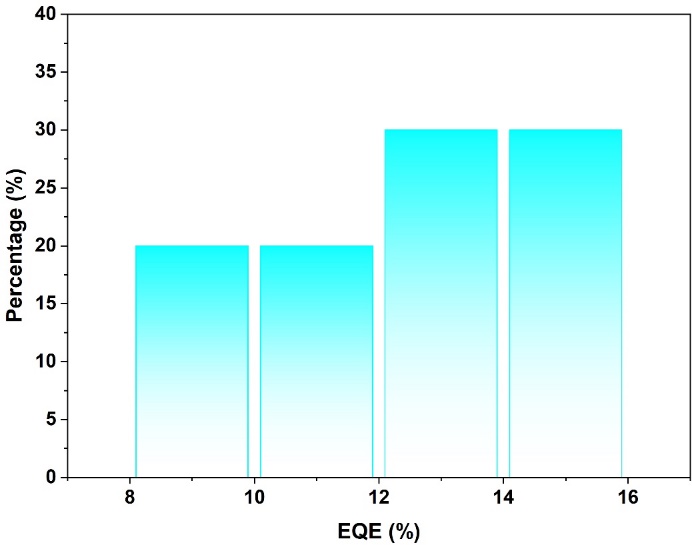


**Fig. S11.** Reproducibility of InP/ZnSe_0.7_S_0.3_/ZnS based QLEDs.

**Table S5.** Performance of state-of-art InP-based green-emitting QLEDs.

| Year | EL peak | Turn-on voltage | Luminance at 4V (cd m^-2^) | Maximum EQE (%) | Phosphorus | Ref. |
| --- | --- | --- | --- | --- | --- | --- |
| 2018 | 532 | 4.5 | <10 | <1 | (TMS_3_)_3_P | 10 |
| 2019 | 530 | 2.8 | 2200 | 6.30 | (TMS_3_)_3_P | 3 |
| 2019 | —— | 2.5 | 1000 | 13.60 | —— | 4 |
| 2020 | —— | 2.4 | 300 | 10.00 | —— | 6 |
| 2020 | 545 | 4.5 | 100 | 1 | (TMS_3_)_3_P | 5 |
| 2021 | —— | 2.5 | 1000 | 1.68 | (DMA)_3_P | 8 |
| 2021 | 525 | 2.9 | <1000 | 7.06 | (DMA)_3_P | 9 |
| 2021 | 545 | 2.2 | 4000 | 16.3 | —— | 11 |
| 2021 | 548 | 2.2 | 1692 | 5.42 | —— | 12 |
| 2022 | 532 | 2.2 | 2300 | 15.2 | (DMA)_3_P | This work |

**Note:** The QDs of Ref. 4, 6,11,12 are offered by other institutions and the phosphorus cannot be identified.

**References**

1 Wang, L. *et al.* Mg-doped ZnO nanoparticle films as the interlayer between the ZnO electron transport layer and InP quantum dot layer for light-emitting diodes. *The Journal of Physical Chemistry C* **124**, 8758-8765 (2020).

2 Kim, Y. *et al.* Bright and uniform green light emitting InP/ZnSe/ZnS quantum dots for wide color gamut displays. *ACS Applied Nano Materials* **2**, 1496-1504 (2019).

3 Zhang, H. *et al.* High‐efficiency green InP quantum dot‐based electroluminescent device comprising thick‐shell quantum dots. *Advanced Optical Materials* **7**, 1801602 (2019).

4 Moon, H. *et al.* Composition-tailored ZnMgO nanoparticles for electron transport layers of highly efficient and bright InP-based quantum dot light emitting diodes. *Chemical Communications* **55**, 13299-13302 (2019).

5 Shin, D. W. *et al.* Water-proof flexible InP@ZnSeS quantum dot light-emitting diode. *Advanced Optical Materials* **8**, 1901362 (2020).

6 Iwasaki, Y., Motomura, G., Ogura, K. & Tsuzuki, T. Efficient green InP quantum dot light-emitting diodes using suitable organic electron-transporting materials. *Applied Physics Letters* **117**, 1801602 (2020).

7 Jo, J.-H. *et al.* InP-Based Quantum Dots Having an InP Core, Composition-gradient ZnSeS inner shell, and ZnS outer shell with sharp, bright emissivity, and blue absorptivity for display devices. *ACS Applied Nano Materials* **3**, 1972-1980 (2020).

8 Jiang, W., Choi, Y. & Chae, H. Efficient green indium phosphide quantum dots with tris(dimethylamino)-phosphine phosphorus precursor for electroluminescent devices. *Journal of Materials Science: Materials in Electronics* **32**, 4686-4694 (2021).

9 Liu, P. *et al.* Green InP/ZnSeS/ZnS core multi‐shelled quantum dots synthesized with aminophosphine for effective display applications. *Advanced Functional Materials* **31**, 2008453 (2021).

10 Ramasamy, P., Ko, K.-J., Kang, J.-W. & Lee, J.-S. Two-step “seed-mediated” synthetic approach to colloidal indium phosphide quantum dots with high-purity photo- and electroluminescence. *Chemistry of Materials* **30**, 3643-3647 (2018).

11 Chao, W.-C. *et al.* High efficiency green InP quantum dot light-emitting diodes by balancing electron and hole mobility. *Communications Materials* **2**, 96 (2021).

12 Guo, S. *et al.* Boosting efficiency of InP quantum dots-based light-emitting diodes by an In-doped ZnO electron transport layer. *IEEE Electron Device Letters* **42**, 1806-1809 (2021).
