# Supplementary material for: Highly efficient green InP-based quantum dot light-emitting diodes regulated by inner alloyed shell component
Source: Light Sci Appl. 2022 May 30;11:162. doi: 10.1038/s41377-022-00855-z (PMC9151710; doi:10.1038/s41377-022-00855-z)
Supplement: Supplementary file 2 — Confidential Certificate [file 41377_2022_855_MOESM2_ESM.pdf]

文章保密与版权转让证明

承　诺　书

此文章不涉密且不存在造假、抄袭、一稿多投等学术不端行为，特此承诺。

第一（通讯）作者签字：于鹏

2022 年 05 月 13 日

《Light: Science & Applications》编辑部：

我单位 于鹏，曹盛，单玉亮，毕玉和，胡雅绮，曾若生，邹炳锁，王允军，赵家龙 作者（需按正式发表文章署名顺序，填写全部作者姓名）为你刊撰写的文章（题目：**Highly efficient green InP-based quantum dot light-emitting diodes regulated by inner alloyed shell component**），经审查，未发现该文章存在涉密内容和造假、抄袭、一稿多投等学术不端现象。该文章若存在涉密内容和造假、抄袭、一稿多投等学术不端问题，《发光学报》编辑部无需承担任何责任。该文章一经录用，其数字化复制权、发行权、汇编权及信息网络传播权将转让予《发光学报》编辑部。

导师（课题负责人）签字：赵家龙

单位盖章

广西大学 物理科学与工程技术学院

2022 年 05 月 13 日
